# Association Between Helicobacter pylori Infection and the Risk of Pancreatic Cancer: A Systematic Review Based on Observational Studies

**DOI:** 10.7759/cureus.28543

**Published:** 2022-08-29

**Authors:** Venkatesh Panthangi, Adrienne R Cyril Kurupp, Anjumol Raju, Gaurav Luthra, Mahrukh Shahbaz, Halah Almatooq, Paul Foucambert, Faith D Esbrand, Sana Zafar, Safeera Khan

**Affiliations:** 1 Internal Medicine, California Institute of Behavioral Neurosciences & Psychology, Fairfield, USA; 2 Pediatrics, California Institute of Behavioral Neurosciences & Psychology, Fairfield, USA; 3 Dermatology, California Institute of Behavioral Neurosciences & Psychology, Fairfield, USA

**Keywords:** pancreatic neoplasm, pancreatic malignancy, pancreatic cancer, h. pylori, helicobacter pylori

## Abstract

*Helicobacter pylori *(*H. pylori*) bacterial infection has long been scrutinized as one of the potential risk factors for the development of pancreatic cancer with quite inconsistent and unequivocal data. Little is known about the risk factors involved with this malignancy. In this systematic review, we aimed to examine the relationship between *H. pylori* infection and pancreatic cancer based on the evidence from the existing observational studies across the world. We searched major electronic databases such as PubMed, MEDLINE, Science Direct, and Cochrane Library. After a careful and thorough screening process, we selected 15 observation studies for this systematic review. Six of 15 studies found a significant association between *H. pylori* infection and pancreatic cancer. Additionally, four of these studies found a significant relationship between the cytotoxin-associated gene A strain of *H. pylori* and pancreatic cancer. Based on the evidence from the selected studies, a weak association was observed between *H. pylori *infection and cancer of the pancreas, especially in European and Asian populations compared to the North American population. The cross-sectional evidence from the case-control studies only suggests the existence of an association but does not provide substantial evidence of the causative relationship. Further large-scale, prospective cohort studies are warranted in the future to understand this contradictory relationship better.

## Introduction and background

Pancreatic cancer is one of the most fatal malignancies in the world. Among cancer-related deaths, pancreatic cancer is currently the seventh leading cause worldwide [1]. According to the statistical data of the American Cancer Society, it is presently the third leading cause of cancer mortality in the United States (US) [2]. The five-year survival rate is about 6%, with a varying range (2%-9%) across the world [3]. The incidence and mortality rates also vary based on geographical locations, with higher percentages in developed countries (Northern America and Western Europe) compared to developing countries (Asia and Africa) [4]. In the last 25 years, pancreatic cancer incidence and mortality rates have increased by more than 50% [5]. By 2040, it could surpass colorectal cancers and become the second most frequent cause of cancer mortality in the US [6]. The reasons for this increasing trend of incidence and mortality rates are yet unclear. Epidemiologists have investigated several risk factors for pancreatic cancer in the past, but still, there is a lot to understand regarding the underlying mechanisms of these factors. Prior studies have identified smoking, old age, male sex, diabetes mellitus, chronic pancreatitis, and family history of pancreatic cancer as possible risk factors [7]. These factors only partially explain the increasing incidence and prevalence of pancreatic neoplasm. Among the modifiable risk factors, the etiopathological role of *Helicobacter pylori *(*H. pylori*) infection has long been investigated with inconsistent findings.

*H. pylori* infection is globally prevalent, affecting approximately more than half of the global population [8]. In North America, the prevalence of *H. pylori* infection is about 35%, with a contrasting disparity of 75% in the indigenous Alaskan population [9]. This Gram-negative organism was recognized as a group I carcinogen by the International Agency for Research on Cancer (IARC) [10]. Numerous clinical studies have identified it as an etiological agent for gastric diseases such as chronic gastritis, peptic ulcer disease, gastric adenocarcinoma, and mucosa-associated lymphoid tissue (MALT) lymphoma of the stomach [11]. *H. pylori* bacteria has also been implicated in several extra-gastric manifestations such as iron deficiency anemia, idiopathic thrombocytopenic purpura, and vitamin B 12 deficiency [12,13]. Due to the proven oncogenic potential of *H. pylori* bacteria with gastric cancer, its association with the etiopathogenesis of pancreatic cancer has become a topic of interest for researchers over the past two decades. Globally, several observational studies evaluated the relationship between *H. pylori* infection and pancreatic carcinoma. Most of these studies were case-control studies, while a few of them were cohort studies. Most researchers measured the seroprevalence of *H. pylori* antibodies to demonstrate the association. Some studies simultaneously measured the serostatus of cytotoxin-associated gene A (CagA), a virulent strain of *H. pylori* that was known for its pathogenesis in various gastric disorders. Interestingly, this CagA protein was not present in all strains of *H. pylori* [14]. Few meta-analyses were also conducted across the world to investigate this association. The results of these earlier observational studies and meta-analyses were inconclusive and unequivocal. Nearly half of the studies had conflicting findings with the other half. Interestingly, one meta-analysis by Wang et al. reported *H. pylori* infection as a protective factor against pancreatic cancer in Eastern populations [15].

Even after numerous studies, there is no conclusive evidence until now about the potential role of *H. pylori* infection in the development of pancreatic cancer. The lack of proper screening modalities, limited treatment options, and poor survival outcomes highlight the critical need to develop an in-depth understanding of the etiopathology of this deadly disease. The purpose of this systematic review is to qualitatively analyze the literature on the association between *H. pylori* infection and the risk of developing pancreatic cancer.

## Review

Methods

We conducted the literature search for this systematic review according to the Preferred Reporting Items for Systematic Reviews and Meta-Analyses (PRISMA) 2020 guidelines [16].

Data Sources and Search Strategy

We used electronic databases such as PubMed, Medical Literature Analysis and Retrieval System Online (or MEDLINE), Science Direct, and Cochrane Library databases for literature search on April 7, 2022. We used keywords "*Helicobacter pylori* AND Pancreatic cancer", and medical subject heading (MeSH) terms ("*Helicobacter pylori*"(Mesh)) AND ("Pancreatic Neoplasms/etiology"(Mesh) OR "Pancreatic Neoplasms/pathology"(Mesh)) OR "Pancreatic cancer, adult" (Supplementary Concept)). We searched for research articles that discussed any kind of relationship between *H. pylori* infection and pancreatic cancer. Additionally, we performed a manual search in the reference lists of the relevant articles.

*Inclusion and Exclusion ​*​​​​​​*Criteria*

Our inclusion criteria were as follows: (i) retrospective or prospective studies that explored the association between *H. pylori* and pancreatic cancer; (ii) full-text peer-reviewed articles published in the English language until April 2022; (iii) articles that discussed only *H. pylori* species; (iv) papers that studied population of all age groups across the world; (v) no date limit for the studies. We used the following exclusion criteria: (i) review articles, gray and unpublished literature, and non-English articles; (ii) articles that included or discussed species other than *H. pylori*; (iii) papers published as letters to editors, commentaries, case reports, case series; (iv) papers that were meeting/conference abstracts; (v) books and documents.

Study Selection

Two reviewers (VP and PF) conducted the screening of articles independently using the eligibility criteria stated above. After removing duplicates from the initial list, we applied automation tools wherever required to eliminate the ineligible records. The reports were screened by reviewing the titles and abstracts. We retrieved the relevant articles and assessed them for eligibility using quality assessment tools.

Quality Assessment

We selected high-quality studies for this systematic review to avoid the risk of bias. The quality assessment was done by two reviewers (VP and PF) separately using the Joanna Briggs Institute (JBI) critical appraisal tool. The JBI checklist for case-control studies consisted of 10 questions, whereas the checklist for cohort studies had 11 questions. We included only the studies that fulfilled most of the criterion list.

Results

**Figure 1:**
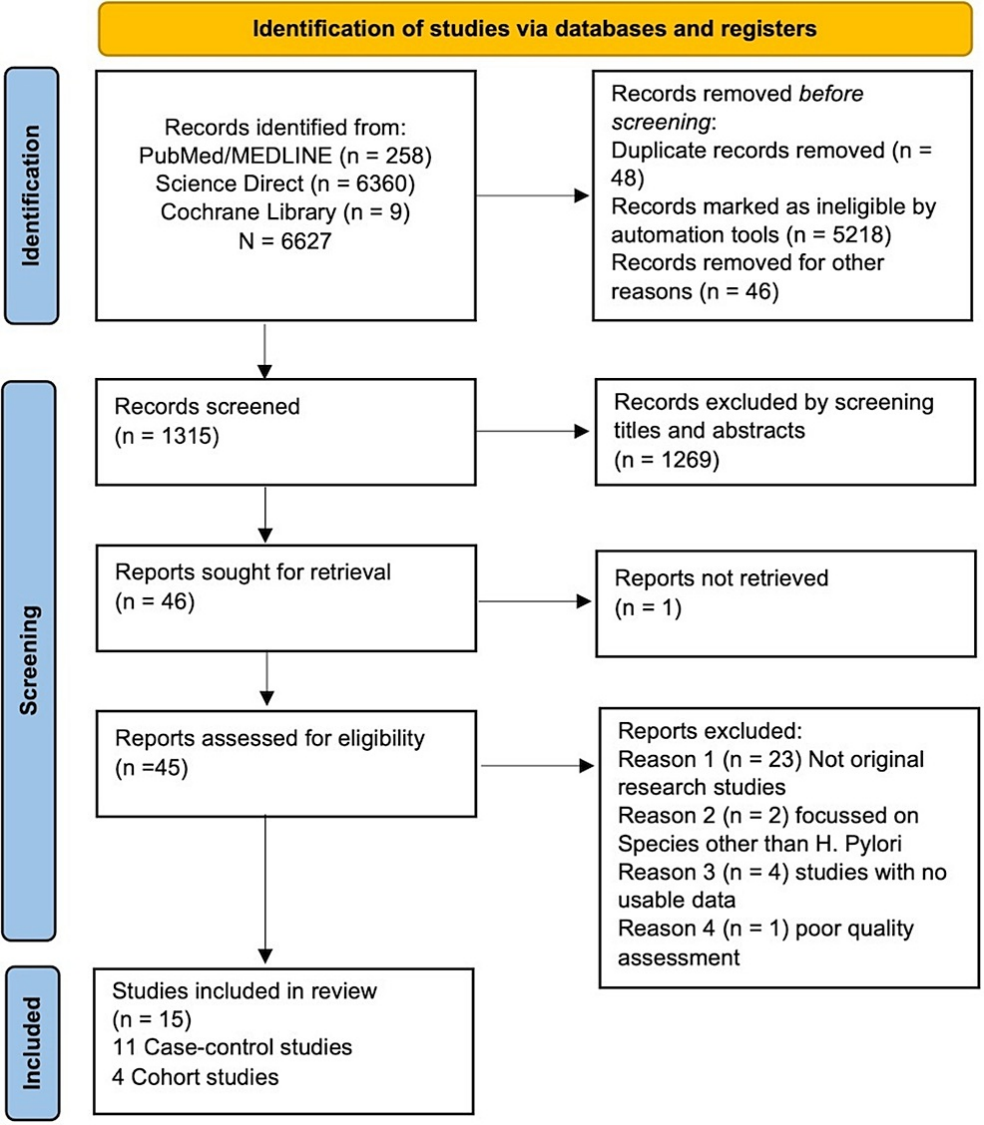
PRISMA 2020 flow diagram showing the study screening and selection process PRISMA: Preferred Reporting Items for Systematic Reviews and Meta-Analyses; *H. pylori*:* Helicobacter pylori*

We identified 6627 articles in our search from electronic databases (PubMed/MEDLINE, Science Direct, and Cochrane). Based on the automation tool criteria, we found 5218 ineligible articles in the Science Direct database. We included only primary research studies using this automation tool. Later, we removed 48 duplicate records and eliminated another 46 reports that were non-English and non-human studies. By reviewing the individual title and abstract, we excluded 1269 articles that were considered unrelated to our topic. Finally, we screened 45 articles thoroughly based on the inclusion/exclusion criteria and quality assessment tools. Among these, we excluded 23 records as they were not original research studies. We also eliminated another four reports as they did not have usable data regarding the association between *H. pylori* and pancreatic cancer. We removed another two studies as they focused on species other than *H. pylori*. Finally, we critically appraised 16 articles using the JBI checklist. Figure 1 summarizes the search strategy and study screening process.

All the finalized reports were observational studies. Hence, we used the JBI critical appraisal tool to assess the quality of these observational studies. Out of the 12 case-control studies appraised critically, we excluded one case-control study due to poor quality. Table 1 summarizes the quality assessment of case-control studies using the JBI checklist.

**Table 1:** JBI critical appraisal of case-control studies JBI: Joanna Briggs Institute List of questions in the JBI critical appraisal checklist: Q1: Were the groups comparable other than the presence of disease in cases or the absence of disease in controls? Q2: Were cases and controls matched appropriately? Q3: Were the same criteria used for the identification of cases and controls? Q4: Was exposure measured in a standard, valid and reliable way? Q5: Was exposure measured in the same way for cases and controls? Q6: Were confounding factors identified? Q7: Were strategies to deal with confounding factors stated? Q8: Were the outcomes measured in a standard, valid and reliable way for cases and controls? Q9: Was the exposure period of interest long enough to be meaningful? Q10: Was appropriate statistical analysis used?

Author and year	Q1	Q2	Q3	Q4	Q5	Q6	Q7	Q8	Q9	Q10	Overall appraisal
Permuth et al., 2021 [9]	Yes	Yes	Yes	Yes	Yes	Yes	Unclear	Yes	Unclear	Yes	Include
Stolzenberg-Solomon et al., 2001 [17]	Yes	Unclear	Yes	Yes	Yes	Yes	Yes	Unclear	Yes	Yes	Include
de Martel et al., 2008 [18]	Yes	Yes	Yes	Unclear	Yes	Yes	Unclear	Unclear	Yes	Yes	Include
Risch et al., 2010 [19]	Unclear	Yes	No	yes	Yes	Unclear	Yes	Yes	Unclear	Yes	Include
Raderer et al., 1998 [20]	Unclear	No	Yes	Yes	Yes	Yes	Unclear	Yes	Unclear	Yes	Include
Lindkvist et al., 2008 [21]	Yes	Yes	Yes	Yes	Yes	Yes	Yes	Unclear	Yes	Yes	Include
Yu et al., 2013 [22]	Yes	Unclear	Yes	Yes	Yes	Yes	Yes	Yes	Yes	Yes	Include
Huang et al., 2017 [23]	Yes	Yes	Yes	Yes	Yes	Yes	Yes	Yes	Unclear	Yes	Include
Risch et al., 2014 [24]	Yes	Yes	Unclear	Yes	Yes	Unclear	Yes	Yes	Unclear	Yes	Include
Ai et al., 2014 [25]	Unclear	Yes	Yes	Yes	Yes	Yes	Yes	Yes	Unclear	Yes	Include
Laya et al., 2022 [26]	Yes	Yes	Yes	Yes	Yes	Yes	Unclear	Yes	Unclear	Yes	Include

Similarly, we assessed the remaining four cohort studies from the finalized list using the JBI checklist for cohort studies. The four cohort studies appraised were of good quality and included in this review. Table 2 shows the results of the quality assessment of the cohort studies.

**Table 2:** JBI critical appraisal of cohort studies JBI: Joanna Briggs Institute List of questions in the JBI critical appraisal checklist: Q1: Were the two groups similar and recruited from the same population? Q2: Were the exposures measured similarly to assign people to both exposed and unexposed groups? Q3: Was the exposure measured in a valid and reliable way? Q4: Were confounding factors identified? Q5: Were strategies to deal with confounding factors stated? Q6: Were the groups/participants free of the outcome at the start of the study (or at the moment of exposure)? Q7: Were the outcomes measured in a valid and reliable way? Q8: Was the follow-up time reported sufficient to be long enough for outcomes to occur? Q9: Was follow-up complete, and if not, were the reasons to loss to follow-up described and explored? Q10: Were strategies to address incomplete follow-up utilized? Q11: Was appropriate statistical analysis used?

Author and year	Q1	Q2	Q3	Q4	Q5	Q6	Q7	Q8	Q9	Q10	Q11	Overall appraisal
Kumar et al., 2020 [27]	Unclear	Unclear	Yes	Yes	Yes	Yes	Yes	Yes	Yes	Unclear	Yes	Include
Chen et al., 2016 [28]	Yes	Yes	Yes	Yes	Unclear	Unclear	Yes	Yes	Yes	Unclear	Yes	Include
Hirabayashi et al., 2019 [29]	Yes	Yes	Yes	Yes	Unclear	Yes	Yes	Unclear	Yes	Unclear	Yes	Include
Hsu et al., 2014 [30]	Yes	Yes	Yes	Yes	No	Yes	Yes	Unclear	Yes	Unclear	Yes	Include

Description of Selected Case-Control Studies

Out of the final 11 case-control studies, six were population-based, and five were nested case-control studies. Altogether, this review included a total of 2395 cases and 3123 controls. The population researched displayed marked diversity in terms of geographical locations and ethnicity. Among these 11 studies, four studies by Stolzenberg-Solomon et al., de Martel et al., Risch et al., and Permuth et al. were from the USA [9,17-19]. Another four studies by Raderer et al., Lindkvist et al., Yu et al., and Huang et al. were from European countries [20-23]. The remaining three studies by Risch et al., Ai et al., and Laya et al. were on the Asian population [24-26]. Table 3 outlines the characteristics of these case-control studies.

**Table 3:** Characteristics of the selected case-control studies *H. pylori*: *Helicobacter pylori*; CagA: cytotoxin-associated gene A; NR: not reported; CI: confidence interval

Author and year	Country of study	Enrollment period	Cases				Controls				Mean age (years)	Males	Odds ratio, *H. pylori* (95% CI)
			No.	H. pylori+	H. pylori-	CagA+	No.	H. pylori+	H. pylori-	CagA+			
Raderer et al., 1998 [20]	Austria	1974-1999	92	60	32	NR	27	12	15	NR	58 vs 56	65 (54.6%)	2.1 (1.1-4.1)
Stolzenberg-Solomon et al., 2001 [17]	Finland	1985-1988	121	99	22	73	226	165	61	115	64 (50-76)	347 (100%)	1.87 (1.05-3.34)
de Martel et al., 2008 [18]	USA	1964-1969	104	51	53	33	262	155	107	83	71.5 +/- 9.7	181 (49.5%)	0.85 (0.49-1.48)
Lidkvist et al., 2008 [21]	Sweden	1974-1999	87	39	48	NR	263	100	163	NR	60.7	245 (70%)	1.25 (0.75-2.09)
Risch et al., 2010 [19]	USA	2005-2009	373	80	293	55	690	120	570	108	68.3 vs 66.9	605 (56.9%)	1.34 (0.94-1.92)
Yu et al., 2013 [22]	Finland	1985-1999	353	325	28	NR	353	328	25	NR	69	706 (100%)	0.86 (0.49-1.51)
Risch et al., 2014 [24]	China	2006-2011	761	233	528	442	794	327	467	537	64.9	895 (57.5%)	0.62 (0.50-0.77)
Ai et al., 2014 [25]	China	2012-2013	56	36	20	14	60	28	32	6	56.8 +/-6.75	73 (62.9%)	NR
Huang et al., 2017 [23]	10 European countries	1992-2000	448	196	250	140	448	206	241	138	57.8 +/- 7.8	426 (47.5%)	0.91 (0.68-1.21)
Permuth et al., 2021 [9]	USA	2004-2015	131	13	118	14	131	16	115	15	67.6 vs 59.0	132 (50.4%)	0.59 (0.25-1.40)
Laya et al., 2022 [26]	India	NR	61	48	13	43	94	72	22	60	55.85 +/- 11.81	99 (63.9%)	NR

Most of these studies measured immunoglobulin G (IgG) antibodies against *H. pylori* using the enzyme-linked immunosorbent assay (ELISA) method to determine the presence of infection. Moreover, eight selected studies simultaneously measured antibodies to CagA+ and CagA- strains of *H. pylori* and investigated their association with pancreatic cancer separately. Among these case-control studies, four studies found a significant harmful association [17,19,20,25]. In contrast, six studies found no statistically significant association between *H. pylori* infection and pancreatic cancer [9,18,21-23,26]. Risch et al. found a decreased risk of pancreatic cancer with the CagA seropositive strain of *H. pylori* [24]. Table 4 outlines the selected case-control studies’ methodology, key findings, and clinical implications.

**Table 4:** Summary of the key findings of selected case-control studies *H. pylori*: *Helicobacter pylori*; CagA: cytotoxin-associated gene A; PC: pancreatic cancer; OR: odds ratio; CI: confidence interval; Ig: immunoglobulin; ELISA: enzyme-linked immunosorbent assay

Author and year	Methodology	Key findings and clinical implications	Overall association
Raderer et al., 1998 [20]	IgG antibodies against *H. pylori* were measured using the ELISA method in histologically confirmed PC cases (n=92) and a control group of healthy volunteers (n=27).	In pancreatic cancer cases, the seropositivity rate of *H. pylori *infection was found to be 65% compared to that of 69% in gastric cancer patients and 47% in normal healthy individuals. The high rate of *H. pylori* infection in pancreatic cancer patients suggested a positive association between both conditions; OR (95% CI) = 2.1 (1.1-4.1).	Harmful
Stolzenberg-Solomon et al., 2001 [17]	IgG levels of* H. pylori* and CagA+ antigen levels in PC cases (n=121) and controls (n=226) were collected using the ELISA method. A conditional logistic regression model was used to calculate the OR and 95% CI.	*H. pylori* seroprevalence was 82% in 121 cases of PC, whereas the rate was 73% in the control group with 226 subjects. The findings of this study suggested a possible role of *H. pylori* infection in the development of pancreatic cancer; OR (95% CI), for *H. pylori* = 1.87 (1.05-3.34); OR (95% CI), for CagA+ = 2.01 (1.09-3.70).	Harmful
de Martel et al., 2008 [18]	The sera of 104 cases of pancreatic cancer were compared with that of 262 controls for antibodies of* H. pylori* and CagA protein.	There was no statistically significant association between *H. pylori *infection and pancreatic cancer in a multivariate analysis both in terms of* H. pylori *antibodies and CagA protein.	No association
Lindkvist et al., 2008 [21]	*H. pylori* serology was analyzed in 87 cases of pancreatic cancer and 262 controls using logistic regression.	No association was found between *H. pylori *infection and pancreatic cancer.	No association
Risch et al., 2010 [19]	ABO blood group and seropositivity for *H. pylori* and CagA protein were determined using the ELISA method in cases (n=373) and matched controls (n=790) to examine the risk of pancreatic cancer.	An increased risk of pancreatic cancer was found in association with the non-O blood group and CagA negative strain of *H. pylori* infection; OR (95% CI) = 1.34 (0.94-1.92).	Harmful
Yu et al., 2013 [22]	Fifteen different *H. pylori*-specific antibodies were determined in an equal number of cases and controls (n=353) using a multiplex serology assay.	Conditional logistic regression analysis was used to measure the OR and 95% CIs. Neither the targeted antigens nor the combination of all the antigens showed any positive association with the risk of pancreatic cancer. This study’s findings suggest that *H. pylori* infection is not a risk factor for the cancer of the pancreas.	No association
Risch et al., 2014 [24]	Seropositivity for *H. pylori* antibodies and its virulent protein CagA protein was obtained from cases (n=761) and matched controls (n=794) using the ELISA method to examine the risk of pancreatic cancer.	Both *H. pylori* antibody and CagA seropositivity serostatus were found to have a decreased risk of pancreatic cancer, whereas CagA-negative *H. pylori *seropositivity showed an increased risk to some extent.	Bidirectional association
Ai et al., 2015 [25]	*H. pylori*-specific IgG, IgM antibodies, and CagA-*H. pylori* IgG antibodies were measured from cases (n=56) and controls (n=60).	The *H. pylori *and CagA seropositivity rate was found to be higher in the observation group. This study’s results indicate that patients with *H. pylori *infection, especially with CagA serostatus, are at an increased risk of pancreatic cancer.	Harmful
Huang et al., 2017 [23]	Seroprevalence of *H. pylori* and CagA antibodies was measured in an equal number of cases and controls (n=448) to investigate the altered risk of pancreatic cancer with *H. pylori* infection.	The results showed that neither *H. pylori* nor CagA serostatus was significantly associated with an increased pancreatic cancer risk.	No association
Permuth et al., 2021 [9]	Fifteen antibodies to *H. pylori*-specific proteins were measured using the multiplex serology method in an equal number of PC cases (n=131) and controls (n=131) to study the risk of PC.	There was no significant association found between the seroprevalence of *H. pylori*-specific proteins overall and pancreatic cancer. The prevalence of a few of these proteins increased the odds of PC, and a few others decreased the odds of PC. But these results were found to be non-significant.	No association
Laya et al., 2022 [26]	IgG antibodies of *H. pylori* and CagA antibodies were measured in PC cases (n=61) and the control group (n=94). The primary objective of this study was to determine the association between *H. pylori* infection and periampullary and pancreatic cancer.	There was no significant association between *H. pylori *and CagA seropositivity of the study group and the control group with PC.	No association

Description of Selected Cohort Studies

We identified two retrospective and prospective cohort studies investigating the association between *H. pylori* infection and pancreatic cancer. Out of the four studies, one study by Kumar et al. was from the USA, another study by Chen et al. was from Germany, one study by Hirabayashi et al. was from Japan, and the other study by Hsu et al. was from Taiwan [27-30]. Table 5 illustrates the general information of these four cohort studies.

**Table 5:** Characteristics of the selected cohort studies HP: *Helicobacter pylori*; NR: not reported; HR: hazards ratio; CI: confidence interval

Author and year	Country of study	Enrollment period	Cohort group	Males	HR (95% CI)
Hsu et al., 2014 [30]	Taiwan	2000-2009	HP group: 6022; comparison group: 24,088	3294 (54.7%); 16,470 (54.7%)	2.77 (1.04-7.39)
Chen et al., 2016 [28]	Germany	2000-2002	9506	4291 (45.1%)	1.32 (0.73-2.39)
Hirabayashi et al., 2019 [29]	Japan	1994-2010	20,116	7316 (36.4%)	0.76 (0.49-1.17)
Kumar et al., 2020 [27]	USA	1994-2018	103,595	101,523 (92%)	NR

Of these four studies, only one study by Hsu et al. 2014 demonstrated a significant harmful association [30]. However, the remaining three studies found no association between infection of *H. pylori* and pancreatic carcinoma [27-29]. Table 6 summarizes the key results of these four cohort studies.

**Table 6:** Summary of the key findings of selected cohort studies HP: *Helicobacter pylori*; CagA: cytotoxin-associated gene A; PC: pancreatic cancer; HR: hazards ratio; CI: confidence interval; AG: atrophic gastritis; *H. pylori*: *Helicobacter pylori*

Author and year	Methodology	Key findings and clinical implications	Overall association
Hsu et al., 2014 [30]	An HP cohort group (n=6022) was compared to a comparison cohort group (n=24,088) to investigate the relationship between *H. pylori* infection and pancreatic cancer risk.	The HP cohort group was found to be at higher risk of colorectal, stomach, and pancreatic cancers compared to the comparison group. The results suggest that HP infection could be an independent carcinogenic risk factor; HR and 95% CI for PC = 2.77 (1.04-7.39).	Harmful
Chen et al., 2016 [28]	Serum antibodies against *H. pylori*, CagA strain, and pepsinogen I and II were measured in 9506 men and women and were followed for up to 10.6 years to evaluate cancer development.	There was no association observed between *H. pylori *or CagA seropositivity and PC risk. Increased risks of gastric cancer were found with AG and CagA+ status.	No association
Hirabayashi et al., 2019 [29]	Serological status of HP infection and AG status were measured in a cohort group (n=20,116) in Japan to investigate the risk for PC.	HP seropositivity and AG status, either individually or in combination, were not found to have a statistically significant association with an increased risk of PC in the Japanese population.	No association
Kumar et al., 2020 [27]	A cohort group of 103,595 patients with HP diagnosis was followed until time to event for future PC diagnosis.	Diagnosis of active infection of HP or its eradication was not found to be associated with the development of PC. Increasing age and chronic pancreatitis were found to have a significant association with PC development.	No association

Discussion

The main objective of this systematic review was to investigate the association between *H. pylori* infection and the risk of pancreatic cancer. Across the 15 studies reviewed, six found a harmful association between *H. pylori* infection and pancreatic cancer [17,19,20,24,25,30]. The remaining nine studies found no significant association [9,18,21-23,26-29]. The inconsistency in the results prevailed globally across all three continents (Asia, Europe, and North America) irrespective of the study design. Despite the increasing trend in incidence rates, pancreatic cancer is still rare compared to other malignancies [31]. Hence, the majority of the selected studies had a small sample size. To our knowledge, the latest meta-analysis on this topic was by Liu et al. in 2017 [32]. Despite several studies, there is no breakthrough in determining the definitive etiological role of *H. pylori* infection in pancreatic cancer. In this systematic review, we incorporated data from all the existing literature, including that of the most recent studies, for a better understanding of this ambiguous association.

Risk Factors for Pancreatic Cancer

To date, several modifiable and non-modifiable risk factors for pancreatic cancer are under investigation. The most common modifiable risk factors include smoking, alcohol, obesity, dietary factors, and *H. pylori* infection [1]. Age, sex, ethnicity, blood group, gut microbiota, family history of pancreatic cancer, genetic susceptibility, and diabetes are the most common non-modifiable risk factors [1]. Among these risk factors, cigarette smoking was the most important and established risk factor for pancreatic cancer in multiple studies [33]. Along with smoking, chronic pancreatitis was also identified as a significant risk factor for pancreatic carcinoma, with an estimated 13-fold increased risk [34]. A family history of pancreatic cancer was another vital risk factor, with an increased risk of 80% in patients with at least one first-degree relative with pancreatic adenocarcinoma diagnosis [35].

Similarly, with obesity, a 10% increased risk was identified for every five BMI units with no gender differences in outcomes [36]. Furthermore, the elderly male population, chronic type 2 diabetes mellitus, African American race, and non-O blood group had varying rates of increased risk [37]. Only recently, researchers have started to suspect the role of *H. pylori* infection in the development of cancer of the pancreas. Two-thirds of these risk factors were potentially modifiable [7]. Researchers have hypothesized a complex interaction of these risk factors in developing pancreatic cancer. Yet, data are scarce regarding the underlying mechanisms through which these risk factors induce carcinogenesis in the pancreas.

Potential Oncogenic Mechanisms

Several researchers postulated numerous theories and mechanisms concerning the carcinogenesis of the pancreas. Excess gastric or duodenal acidity and exposure to N-nitroso compounds and their precursors through smoking, dietary, and occupational sources were some of the established theories of pancreatic carcinogenesis [38]. Researchers illustrated two hypothetical pathways for pancreatic cancer based on the location of *H. pylori* colonization in the gastrointestinal tract. One hypothesis involved the concept of excessive gastric acid secretion from the gastric antral colonization of *H. pylori*. Subsequent effects ensue an enhanced duodenal release of secretin with a proportionate bicarbonate output from the pancreas. These effects lead to increased DNA synthesis resulting in pancreatic ductal hyperplasia [38]. The other hypothesis elucidated the concept of decreased gastric acid output from the *H. pylori* colonization of the gastric corpus. This altered acidity was implicated in bacterial overgrowth and excessive secretion of N-nitroso compounds. However, the risk of pancreatic cancer was higher in patients with duodenal ulcers than in gastric ulcers [39].

The gut colonization of *H. pylori* often involves a complex interaction of inflammatory cytokines such as interleukin-1b (IL-1b), tumor necrosis factor-a (TNF-a), and IL-10 [38]. These cytokines can regulate gastric acid production and play an essential role in the inflammatory process [40]. The polymorphism of these cytokine genes significantly correlates with non-cardia gastric cancers in prior research studies [41]. Additionally, *H. pylori* infection was implicated in the hypersecretion of gastric hormones, such as gastrin and somatostatin, known for their proliferative effect on gastric epithelium and pancreatic cancer cell lines [42,43]. Overall, there seems to be a significant resemblance in the underlying mechanisms of gastric and pancreatic oncogenesis, but the latter has the worst outcomes worldwide.

Association of H. pylori Infection and Pancreatic Cancer in the European Population

Across 15 studies reviewed, five case-control studies and one prospective cohort study included the European population. The first case-control study examining the association between *H. pylori* infection and human pancreatic cancer was conducted in 1998 by Raderer et al. in Austria [20]. In this study, the *H. pylori* seroprevalence rates of patients with pancreatic cancer (n=92) were compared to that of patients with gastric adenocarcinoma (positive control; n=35) and colorectal cancer (negative control; n=30) and healthy controls (n=25). High seroprevalence rates were found with gastric cancer (69%) and pancreatic cancer (65%) when compared with colorectal cancer (45%) and healthy subjects (47%). There was no statistically significant difference in the seroprevalence rates between gastric and pancreatic cancer patients. However, there was a substantial difference between individuals with pancreatic cancer, patients with colorectal malignancies, and healthy subjects. This study demonstrated only a positive association but did not elucidate the causal relationship between *H. pylori* infection and pancreatic cancer. The preliminary findings of this study drew much attention from researchers globally.

A nested case-control study by Stolzenberg-Solomon et al. in 2001 reported a similar association in Finland [17]. The subjects for this study were from a cohort group of 29,133 male smokers from Finland who were a part of the Alpha-Tocopherol, Beta Carotene (ATBC) cancer prevention study. Patients diagnosed with pancreatic cancer were identified during a 10-year follow-up period from 1985 to 1995 through the Finnish Cancer Registry. This was the first prospective study that noticed a statistically significant relationship between *H. pylori* infection and pancreatic cancer. A total of 121 cases were matched based on age, study center, date of the blood draw, and trial intervention with 262 control subjects. In addition to the IgG antibodies against *H. pylori*, antibodies of CagA+ protein were also measured simultaneously in this study. Statistical analysis revealed an increased risk of pancreatic cancer in subjects with *H. pylori* or CagA+ strains compared to seronegative individuals.

Compared to the previous study by Raderer et al., this study had a more robust methodology regarding the prospective nature of serum collection (1-10 years), matched control group from the same cohort, and a larger sample size. Stolzenberg-Solomon et al. used the same ATBC study cohort group and conducted another prospective study in 2003 to examine the association between poor dentition history and pancreatic cancer and found a statistically significant association [44]. However, no significant association was found between tooth loss and *H. pylori* seropositivity. One major limitation of this study cohort was that all the subjects were male smokers, which interpreted findings as less generalizable to the non-smoking population.

In contrast to these findings, another nested case-control study conducted by Lindkvist et al. in 2008 in Sweden reported no association between *H. pylori* infection and pancreatic cancer [21]. The cases diagnosed with pancreatic cancer (n=87) were selected from 33,346 residents of Malmo, Sweden, who attended a local wellness screening investigation. Patients were matched using gender, age, and baseline investigation time with control subjects (n=263). Serological analysis of *H. pylori* infection was performed using the ELISA method. They found a significant association in the never-smokers group and a borderline significant association with the low-alcohol-consumption subgroup of the study cohort. However, these subgroups had a minimal number of pancreatic cancer cases making the observation questionable. The overall conclusion of this study goes in line with another case-control study conducted in Germany by Jesnowski et al. 2010 [45]. In this study, they investigated the presence of *H. pylori* DNA sequences in the pancreatic cancer tissue samples, but none of the samples detected the gene sequences. The small sample size (n=65) was the main limitation of this study.

Yu et al. conducted another nested case-control study in 2013 within the same ATBC cohort of Finnish male smokers [22]. Pancreatic cancer cases (n=353) were matched with an equal number of control subjects (n=353) based on age, baseline serum collection date, and follow-up duration. This study used a multiplex serology assay to identify antibodies against 15 *H. pylori*-specific antigens. Individuals who were seropositive to four or more antigens were identified as overall *H. pylori* seropositive. Statistical analysis revealed no significant risk of pancreatic cancer with any of the 15 antigens individually or with overall *H. pylori* seropositivity, defined as the presence of four or more antibodies. Based on these results, Yu et al. proposed that *H. pylori* infection had no role in developing pancreatic cancer. These findings were quite contradictory to those of Stolzenberg-Solomon et al. who used the same patient population from the ATBC study cohort [17]. The main difference in the methodology of both these studies was the use of a multiplex serology assay instead of the ELISA method and the longer follow-up period of approximately 23 years in the study by Yu et al. [22].

The nested case-control study by Huang et al. based on the European Prospective Investigation Into Cancer and Nutrition (EPIC) cohort was the latest study on this topic in the European population [23]. It was a large cohort study that enrolled 520,000 subjects from multiple centers in 10 different European countries (Denmark, France, Germany, Greece, Italy, Norway, Spain, Sweden, the Netherlands, and the United Kingdom). Cases with pancreatic cancer (n=448) were matched individually with control subjects (n=448) using age, gender, study center, time, and fasting status of serum collection as matching factors. The exposure assessment measured *H. pylori* and CagA serostatus using the ELISA method. Serum pepsinogen I and II levels and ABO blood group status were the other biomarkers used in this study. Statistical analysis revealed no association between either *H. pylori* or CagA seropositivity and pancreatic cancer. Further investigation, including the ABO blood group, showed the same results with no significant association. However, they found an increased risk of pancreatic cancer with chronic corpus atrophic gastritis (AG), predominantly with the *H. pylori* seronegative subgroup. This observation warrants confirmation with further investigation.

Chen et al. published a population-based prospective cohort study of the association of *H. pylori* and chronic AG with gastric, pancreatic, and colon carcinoma [28]. The study included 9506 participants, including men (n=4291) and women (n=5215) aged 50-75 years from Saarland, Germany. Serostatus of antibodies against general *H. pylori* and CagA strain of *H. pylori* were collected using the ELISA method. Serum pepsinogen I and II levels were measured using the same ELISA method to determine the AG status. During the 10-year follow-up period, 27 gastric cancer, 46 pancreatic cancer, and 108 colonic incident cancer cases were registered in the Saarland Cancer Registry. Statistical analysis showed no association of *H. pylori* infection with either colonic or pancreatic cancers, irrespective of CagA and AG serostatus. However, the presence of both AG and CagA+ serostatus was strongly associated with the risk of non-cardia gastric cancers. This study was the first and one of the most significant prospective cohort studies conducted on the European population. Out of the six European studies reviewed above, only two [17,20] identified a significant association between *H. pylori* infection and the risk of pancreatic cancer.

Association of H. pylori Infection and Pancreatic Cancer in the North American Population

In this review article, we identified three case-control studies and one retrospective cohort study conducted in the USA. To our knowledge, de Martel et al. were the first group of researchers in the USA to conduct a nested case-control study on this association [18]. They selected the participants for this study from a group of 128,992 members enrolled in routine health check-ups at Kaiser Permanente Medical Care group. This study included pancreatic cancer cases (n=104) and control group subjects (n=262) free of pancreatic cancer who were matched individually based on age, sex, race, and date of health check-up. Serum antibodies of *H. pylori* and CagA protein were measured in both groups. The results showed no significant association in the development of pancreatic cancer either with *H. pylori* or with CagA protein. This study suggests that *H. pylori* infection is not a risk factor for pancreatic cancer. The strengths of this study were the large sample size, the random selection of cases from incident pancreatic cancer cases in the cohort group, and the diverse patient population. Nevertheless, there were a few limitations, such as checking the *H. pylori* status only at the beginning stage of the process and using two different ELISA tests for other control groups, which could have resulted in false-positive rates. Despite these limitations, the authors support their conclusion on the null association.

In 2010, Risch et al. published a study investigating the risk of pancreatic cancer with the ABO blood group, *H. pylori* seropositivity, and the virulent protein of *H. pylori*, CagA [19]. This population-based case-control study included pancreatic cancer cases (n=373) and control subjects (n=690) matched on age and sex. Serological analysis of *H. pylori* and CagA protein was performed using the ELISA method. The results suggested an increased risk of pancreatic cancer with CagA-negative *H. pylori* seropositivity, with an even greater risk, particularly in the non-O blood group individuals. However, there was no significant association with CagA seropositivity. In contrast to these findings, most recently, Permuth et al. published a case-control study including an equal number of matched cases and control subjects (n=131) identified from the Moffitt Cancer Center and Research Center in Tampa, Florida [9]. This study used a multiplex serology assay to measure antibodies against 15 different *H. pylori* antigens. Overall, *H. pylori* seroprevalence was determined when there was reactivity to more than four *H. pylori* proteins. There was no significant association between pancreatic cancer risk and overall *H. pylori* seroprevalence or CagA seropositivity. The contrasting differences in the results of both these studies could be attributed to the method of serological analysis. Permuth et al. used a multiplex serology assay, a more sensitive test compared to the ELISA method used by Risch et al.

In 2020, Kumar et al. conducted a retrospective cohort study examining the controversial association between *H. pylori* infection and pancreatic cancer [27]. This study included a cohort of 103,595 patients in which *H. pylori* diagnosis was determined by pathology, serum antibody, stool antigen, and urea breath testing. Diagnosis of future pancreatic cancer was the primary outcome for this cohort group. A time-to-event analysis revealed no association between active *H. pylori* infection or its eradication with the development of pancreatic cancer. However, secondary analysis in this study showed a significant association between chronic pancreatitis and the risk of pancreatic cancer. The main strength of this study was the population that was the largest cohort of *H. pylori* patients in North America.

Association of H. pylori Infection and Pancreatic Cancer in the Asian Population

In 2014, Risch et al. and Ai et al. separately conducted two case-control studies in China to study this association [24,25]. The study by Risch et al. had a larger sample size of 761 cases and 794 controls compared to 56 patients and 60 control subjects in the study by Ai et al. Both the studies used the ELISA method to measure the serum antibodies against *H. pylori* and its virulent protein CagA. Despite many similarities in the methodology and study models, the results of both these studies were inconsistent. Risch et al. found a decreased pancreatic cancer risk with the CagA seropositivity and a borderline increased risk with the CagA-negative strain. On the other hand, Ai et al. reported a significantly higher percentage of *H. pylori* and CagA-Hp IgG antibodies in the observation group compared to the control group. These findings suggested an increased risk of pancreatic cancer with *H. pylori* infection, particularly with CagA serostatus [25].

During the same period, Hsu et al. conducted a population-based retrospective cohort study in Taiwan to investigate the correlation between *H. pylori* and the cancer risk [30]. In this study, participants selected from a National Health Insurance (NHI) database included a comparison cohort (n=24,088) and an *H. pylori* cohort (n=6022). They calculated the cancer risk in both groups using the multivariable Cox proportional model. They found an increased risk of gastric, pancreatic, and colorectal cancers in the *H. pylori* cohort group as to the comparison cohort. This study suggests that *H. pylori* infection could be an independent carcinogenic risk factor. However, the critical limitation of this study was that they could not adjust the participants for certain variables such as smoking habits, body mass index, alcohol consumption, and socioeconomic status.

Contrary to these findings, Hirabayashi et al. published another population-based prospective cohort study in Japan [29]. This study enrolled men and women (n=20,116) from a local Japan Public Health Center (JPHC) cohort. They measured the *H. pylori* and atrophic gastritis status from the blood samples collected during routine health check-ups. After an approximate 16-year follow-up, they identified 119 pancreatic cancers in this cohort group. They found no significant association of the pancreatic cancer incidence with either *H. pylori* seropositivity or AG status, independently or in combination. This was the first and largest prospective cohort study on the Japanese population to investigate this association. One of the main limitations of this study was that only 36% of the patient population were men. This small sample of the male gender might have caused the results to be gender biased.

In 2022, Laya et al. conducted a case-control study investigating this association in the Indian population [26]. This has been the most recent study to examine this association. This study was a retrospective case-control study based on a single center in India, which included 61 subjects in the study group and 94 participants in the control group. IgG antibodies of *H. pylori* and CagA antibodies were measured using the ELISA method. The primary objective of this study was to examine the risk of periampullary and pancreatic cancer with *H. pylori *infection. The results suggested no significant association between *H. pylori* or CagA antibodies and periampullary/pancreatic cancer. The authors suggest that eradicating this Gram-negative bacterial infection may not be an effective preventive strategy in pancreatic cancer patients. This study was the first to include patients with periampullary cancers in the study group. Nevertheless, there were a few limitations, such as fewer cases in the study group and the presence of advanced-stage disease in the case subjects.

Strengths

One major strength of this systematic review is the large sample size. To the best of our knowledge, this study has analyzed the largest number of pancreatic cancer cases. We selected high-quality observational studies from the beginning until early 2022. Since the most recent meta-analysis in 2017, we were able to identify four more studies (two case-control and two cohort studies) and included those studies in our review for qualitative analysis. The selected studies represented a diverse patient population of various races and ethnicities worldwide.

Limitations of Selected Studies

The authors reported several limitations in their corresponding studies. First, the most common limitation of most studies was the small sample size. Due to its rare occurrence and high mortality rates, the sample size of pancreatic cancer cases was relatively small. Second, the use of serological analysis to determine the *H. pylori* status does not differentiate between past and current infections. As more than 50% of the world’s population is estimated to get *H. pylori* infection at some stage, a significant proportion will have *H. pylori* seropositivity. This ubiquitous seropositivity could lead to a biased interpretation of the association findings. Third, most of the selected studies used the ELISA method to determine *H. pylori* status, a less sensitive test than the multiplex serology assay. Last, there was no consistency in the variables used across the studies for adjusting the samples. This inconsistency might have allowed the confounding factors to jeopardize the reliability of the data.

Limitations of This Systematic Review

There are a few limitations in our systematic review as well. Though high-quality studies were included in our review, two-thirds of included studies were case-control (n=11) compared to cohort (n=4), allowing a higher risk of selection and survival bias. Moreover, we selected the records that were published only in the English language and excluded gray literature. Given the varied trends for the incidence and mortality rates for pancreatic cancer across different continents, the data from non-English publications could have provided more evidence on the underlying mechanisms of this ambiguous association.

Implications and Future Directions

Although the study results were inconsistent, the significant association found between *H. pylori* infection and pancreatic cancer risk in some studies could not be ignored. The increasing trends in the incidence and mortality rates of pancreatic cancer have become quite a challenge in oncology. There is a great need to conduct future studies with robust methodology to identify all possible risk factors behind this increasing incidence rate of pancreatic cancer. As the serological prevalence of *H. pylori *only determines the temporal association with pancreatic cancer, future studies should consider standardized diagnostic testing such as biopsy or stool-based studies. Furthermore, the effect of treated versus untreated *H. pylori *infections on the incidence of pancreatic cancer need to be explored to determine a significant causative association. Moreover, there has been a rising trend in hospitalizations among pancreatic cancer patients in the last decade, adding more burden to the healthcare system [46]. To date, all the existing studies have only investigated *H. pylori* infection as a risk factor, but none examined the prognostic effects of pancreatic cancer with this infection. More than 90% of newly diagnosed pancreatic cancer patients die in the first five years [47]. There is a significant gap in knowledge in understanding the reasons behind this grim prognosis. Future research should include variables and exposures that investigate the risk factors and examine the prognosis of pancreatic cancer.

## Conclusions

In this systematic review, we aimed to untangle the ambiguous relationship between *H. pylori* infection and the risk of pancreatic cancer. We selected high-quality observational studies on a diverse patient population in multiple institutions worldwide. Only six out of 15 studies found a significant association between *H. pylori* infection and the risk of pancreatic cancer. Among these six studies, five included the European and Asian populations, and only one study included the North American population. However, this weak association offered no conclusive evidence of the oncogenic potential of *H. pylori* infection. Nevertheless, there is a critical need to understand the complex interactions between various modifiable and non-modifiable risk factors of pancreatic cancer. For that reason, future research should focus on well-designed, large-scale, prospective cohort studies with more extended follow-up periods. Given the lack of proper screening methods and limited treatment options for pancreatic cancer, a better and complete understanding of the risk factors and underlying mechanisms offers a unique opportunity to prevent this deadly malignancy.
